# The 18-electron rule for main-group alkaline earth octacarbonyl complexes

**DOI:** 10.1093/nsr/nwy129

**Published:** 2018-11-10

**Authors:** Xueming Yang

**Affiliations:** Dalian Institute of Chemical Physics, Chinese Academy of Sciences and Department of Chemistry, Southern University of Science and Technology, China

Elements in the periodic table are classified into s- and p-block main-group elements, d-block transition metals and f-block lanthanides and actinides according to the valence atomic orbitals (AOs). Main-group elements form bonds using their *n*s and *n*p valence orbitals, following the octet rule—a well-known chemical rule which states that atoms tend to bond in such a way that each atom has a closed-shell 18-electron configuration like noble gases. Besides one *n*s and three *n*p valence orbitals, transition metals have additional five *(n-1)*d orbitals. This leads to the 18-electron rule.

Transition metal carbonyl complexes known since the mid-nineteenth century are archetypical examples for illustrating the 18-electron rule. The bonding interactions between carbon monoxide and the metal center can be described using the Dewar−Chatt−Duncanson model, which involves synergic σ donation of the outermost lone-pair 5σ electrons of CO into an empty orbital of the metal and π back donation of electron density from the transition metal d_π_ orbitals into the lowest unoccupied π* anti-bonding orbitals of CO. The π back donation weakens the C–O bond and causes a red shift of the carbonyl stretching frequency, which can be confirmed spectroscopically. Ni(CO)_4_, Fe(CO)_5_ and Cr(CO)_6_ are textbook examples of stable 18-electron complexes. Recent studies [1,2] by Mingfei Zhou from Fudan University and Gernot Frenking from Nanjing Tech University and the University of Marburg demonstrated on the carbonyl complexes of alkaline earth elements that the 18-electron rule and d orbital bonding are not only applicable to transition metals, but can also be extended to main-group heavier alkaline earth elements. This work has just been published in the *Science* magazine [2].

**Figure 1.**
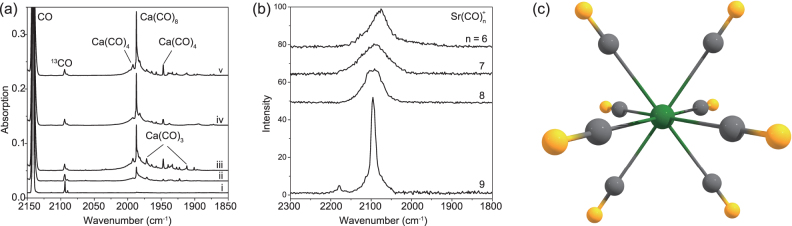
(a) Infrared absorption spectra of calcium–carbonyl complexes from co-deposition of laser-evaporated calcium atoms with 0.1% CO in neon. Spectra measured: (i) after 30 min of sample deposition at 4 K, (ii) after a 12-K annealing period, (iii) after a 13-K annealing period, (iv) after 15 min of visible light irradiation and (v) after another 12-K annealing period. (b) Infrared photodissociation spectra of the Sr(CO)*_n_*^+^ (*n* = 6 to 9) complexes. (c) The structure of the M(CO)_8_ (M = Ca, Sr, Ba) complexes. Adapted from [2].

**Figure 2.**
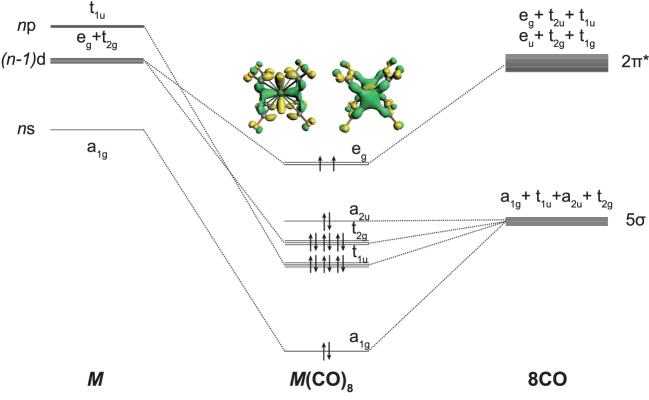
Bonding scheme of M(CO)_8_ (M = Ca, Sr, Ba). Splitting of the *spd* valence orbitals of an atom M in the octacoordinate cubic (*O_h_*) field of eight CO ligands is also given. Only the occupied valence orbitals that are relevant for the M–CO bonding are shown. Adapted from [2].

The alkaline earth elements beryllium, magnesium, calcium, strontium and barium, with an *n*s^2^ valence-shell configuration, typically engage in chemical bonding as ionic salt compounds or in polar bonds via their two *n*s valence electrons. Alkaline earths are in their +2 oxidation state. For the heavier alkaline earth atoms, calcium, strontium and barium, the *(n-1)*d AOs are empty and lie not very far above the *n*s orbital, particularly for the metal cations. Earlier studies have already suggested that the heavier alkaline earth elements can employ their *(n-1)*d AOs to some extent in chemical bonds in some exotic systems [3,4]. Mingfei Zhou and colleagues prepared barium carbonyl complexes in a low-temperature neon matrix and analysed the complexes using infrared absorption spectroscopy. The monocarbonyl cation and anion of barium were formed in the experiments using low CO concentrations, which show a strong red shift of the carbonyl stretching mode with respect to free CO [1]. A theoretical analysis by Gernot Frenking and coworkers confirmed that the barium center was using predominately 5d orbitals instead of 6s and 6p orbitals in bonding with CO. The researchers then prepared the coordinatively saturated carbonyl complexes in a neon matrix. It was surprising to find that not only barium, but also strontium and calcium form octacarbonyl M(CO)_8_ complexes, which are characterized to have cubic O_h_ symmetric structures based on infrared spectroscopic studies in a matrix (see Fig. 1). Simple electron counting indicates that these octacarbonyls fulfill the 18-electron rule. Theoretical calculations confirmed that the octacarbonyls have a cubic (*O_h_*) equilibrium geometry and a triplet (^3^A_1g_) electronic ground state with a triplet reference state and (*n*)s^0^(*n*-1)d^2^ electron configuration of the metal center. The metal center has a zero formal oxidation state. A detailed analysis of the electronic structure (see Fig. 2) indicates that a doubly occupied molecular orbital with a_2u_ symmetry is purely ligand-based because there is no valence AO of the metal that possesses this symmetry. The two valence electrons of metal occupy the degenerate *(n-1)*d orbitals (with e_g_ symmetry) that engage in strong π back-donation interaction with the CO ligands that accounts for the strong red shift of the C–O stretching frequencies. Since two valence electrons of the ligands are not available for donation to the central metal atom in the cubic octacarbonyl complexes, a complete saturation of the valence shell requests 20 valence electrons. This has recently been shown in the transition metal octacarbonyl anions [TM(CO)_8_]^−^ (TM = Sc, Y, La) [5]. Since only 18 electrons are available in M(CO)_8_ (M = Ca, Sr, Ba), the degenerate e_g_ is occupied by two electrons with the same spin giving a triplet (^3^A_1g_) electronic ground state.

The new results challenge previous notions of coordination chemistry and expand our understanding of the group 2 chemistry. The heavier alkaline earth elements appear to have much richer chemistry than hitherto thought. The study foreshadows additional complexes with very interesting bonding motifs mimicking transition metal complexes.
